# BM-MSCs promote prostate cancer progression via the conversion of normal fibroblasts to cancer-associated fibroblasts

**DOI:** 10.3892/ijo.2015.3060

**Published:** 2015-06-22

**Authors:** SIMENG WEN, YUANJIE NIU, SHUYUAN YEH, CHAWNSHANG CHANG

**Affiliations:** 1 Chawnshang Chang Sex Hormone Research Center, Tianjin Institute of Urology, Tianjin Medical University, Tianjin 300211, P.R. China; 2 George Whipple Lab for Cancer Research, Departments of Pathology and Urology, University of Rochester Medical Center, Rochester, NY 14642, USA; 3 Sex Hormone Research Center, Taichung Medical University. Taichung 404, Taiwan, R.O.C.

**Keywords:** bone marrow derived mesenchymal stem cell, cancer associated fibroblast, prostate cancer, TGFβ-1

## Abstract

Recent studies have suggested that prostate cancer (PCa) is able to recruit bone marrow derived mesenchymal stem cells (BM-MSCs) to promote metastasis. The detailed mechanisms, especially the involvement of stromal cells, remain unclear. We found that the recruited BM-MSCs might be able to convert the normal fibroblasts to more cancer associated fibroblast (CAF)-like characteristics via alteration of secreted TGFβ-1. The consequences of such conversion might then enhance the PCa growth and invasion. Addition of functional TGFβ-1 or interruption with TGFβ-1 inhibitor SB431542 led to alteration of the BM-MSCs-induced CAF conversion and influence on the PCa cell growth and invasion. Together, these results suggest that BM-MSCs not only can be directly recruited by PCa epithelial cells to promote PCa invasion, they can also go through conversion of normal fibroblasts to CAFs to enhance PCa cell growth and invasion. Targeting the infiltrating BM-MSCs via either interruption of the interaction between PCa and BM-MSCs or prevention of the conversion of NFs to CAFs via inhibition of TGFβ-1 signal may result in the suppression of PCa progression.

## Introduction

The majority of identified tumors, including prostate cancer (PCa) were proven to be originated from epithelial cells (1). For many years, tumorigenesis has been classically regarded as a largely cell-autonomous process involving genetically transformed cancer cells. Consequently, the focus of cancer research has been on genetic changes in cancer cells, with the progression from normal to malignant state. Recently, increasing number of studies suggest that the progression of tumors might not only rely on the cell-autonomous process of cancer cells themselves but tumors could be also influenced by the surrounding cells (2). The tumor microenvironment (TME) participation in tumor is now well accepted. Most of the solid tumors are surrounded by complicated microenvironment, including the extracellular matrix (ECM), different stromal cells (smooth muscle cells and fibroblasts), other resident cells and recruited inflammatory cells or bone marrow mesenchymal stem cells (BM-MSCs) (2–4).

The peritumoral fibroblasts are considered as active fibroblasts, which are also known as cancer associated fibroblasts (CAFs) or myofibroblasts. In the normal tissue, fibroblasts can be activated for wound healing or fibrosis (5). The activated myofibroblasts can gain contractile stress fibers by expressing α-smooth muscle actin (α-SMA) (6) that allows them to gain better mobility via stronger contractile and secretory capabilities. Therefore, more and more data suggest that myofibroblasts or CAFs might have the capacity to promote tumor growth and metastasis, either via direct interaction with tumor epithelial cells or via the recruitment of inflammatory cells (7,8).

Recent studies found PCa cells, but not normal prostate cells, could better recruit BM-MSCs and the consequences of such increased recruitment might then lead to enhanced PCa metastasis (9). The involvement of stromal cells in such enhanced PCa metastasis, however, remains unclear. In this study, we focused on the roles of stromal cells in the BM-MSCs capacity to enhance PCa growth and invasion and results revealed that recruited BM-MSCs converted normal fibroblasts (NFs) to more CAF-like cells to further promote PCa cell growth and invasion.

## Materials and methods

### Cell culture

TRAMP-C1, CWR22Rv1, LNCaP, and C4-2 cell lines were obtained from ATCC (Manassas, VA, USA). TRAMP-C1 cells were maintained in Dulbecco’s modified Eagle’s medium with 10% FBS. CWR22Rv1, LNCaP, and C4-2 cells were cultured in RPMI-1640 medium with 10% FBS.

Mouse primary BM-MSCs were isolated from wild-type C57BL/6 mice (Jackson Laboratory, Bar Harbor, ME, USA) as previouly described (10). Tibias and femurs were dissected from 6-week-old mice. After bones were cut, the marrows were flushed out with 5 ml DMEM by using a needle and syringe, and re-suspended in DMEM plus 15% FBS. After filtration, mono-nucleated cells were maintained in DMEM (Gibco) with 15% FBS, 2 mM L-glutamine, 100 U/ml penicillin, 100 mg/ml streptomycin, and 10 mM HEPES. All of the cells were incubated at 37°C with 5% humidified CO_2_ for 24 h, then the non-adherent cells were removed by replacing the medium every 3 days for ~1 week. When the cells grew to confluence, they were harvested with 0.25% trypsin and 1 mM EDTA (Hyclone) for experiments.

Human BM-MSCs were purchased from Stem Cell Technologies (Vancouver, BC, Canada), and maintained in Human MesenCult^®^ Proliferation kit (Stem cell Technologies Inc.). BM-MSCs were already verified by adipogenesis and osteogenesis differentiations in our lab (10). As described previously (11), mouse CAFs was isolated from the prostate stromal region of transgenic adenocarcinoma of the prostate mice in our lab. Mouse NFs were isolated from the prostate stromal region of benign prostate mice in the same way. pshTERT is an immortalized stromal cell line, stably expressing the human telomerase catalytic subunit-hTert (a kind gift from the New York University, named pshTERT). NFs and CAFs were cultured in RPMI-1640 medium. All cells were maintained in a humidified 5% CO_2_ environment at 37°C.

### Cell viability assay (MTT)

TRAMP-C1 cells (10^4^) with different treatment were seeded in 24-well plates, and cultured in 10% FBS media for 0, 2, 4 and 6 days. Cells from different time-points were harvested, then we added 3-(4,5-dimethylthiazol-2-yl)-2,5-diphenyltetrazolium bromide (MTT, 0.5 mg/ml in normal media), incubated for 2 h and removed the media. The cellular staining products were dissolved in 0.5 ml DMSO. The absorbance at OD570 was measured by spectrophotometry (Beckman Du640B). The data represent means ± SD from three independent wells and confirmed by three independent experiments.

### BrdU labeling

Cells (10^3^) were seeded on the 6-well plates, given certain treatment (such as NFs or CAFs) and cultured to 50% confluence. BrdU labeling reagent (Invitrogen) was added to the cultured cells for 1:100 dilution in the culture media for 24 h. After labeling, staining was performed according to the manual instructions (Invitrogen, BrdU staining kit). The data represent means ± SD from three independent wells and confirmed by three independent experiments.

### Invasion assay

The 6-well transwells (Corning, #3422) were coated with growth factor reduced matrix gel diluted with serum-free RPMI-1640 media (1:5 diluted), and dried overnight at 37°C. Cells (10^5^) after different treatments were re-suspended with serum-free media and seeded in the upper transwells. Media with 10% FBS was put in the lower wells. After incubation for 48 h, the cells on the upper surface of the transwells were removed with cotton swabs. Cells on lower filter surfaces were fixed with 75% ethanol, stained with 0.1% toluidine blue in PBS. Invaded cells were counted under a microscope. The data represent means ± SD from three independent wells and confirmed by three independent experiments.

### Western blot analysis

Harvested cells were washed with PBS twice and lysed in RIPA buffer (50 mM Tris-HCl/pH 7.4, 1% NP-40, 150 mM NaCl, 1 mM EDTA, 1 mM Na_3_VO_4_, 1 mM NaF, 1 mM okadaic acid and 1 mg/ml aprotinin, leupeptin, and pepstatin) with proteinase inhibitor. Samples (30 μg protein) were loaded on 10% SDS-PAGE gel to be separated and transferred to PVDF membranes at 4°C (100 V, 90 min). Membranes were blocked in 5% fat-free milk with 3% BSA in PBST for 1 h at room temperature, and incubated with appropriate dilutions of primary antibodies (α-SMA, Abcam #ab7817, 1:300; GAPDH, Santa Cruz #sc-32233, 1:3,000) overnight at 4°C. Then the membranes were washed, and incubated with HRP conjugated anti-mouse IgG for 1 h at room temperature. The blots were developed in ECL mixture and visualized by an imager.

### RNA isolation and quantitative analysis

Total cellular RNAs were extracted by TRIzol reagent (Invitrogen) according to the manufacturer’s instructions. Total RNA (1 ng) was used to synthesize first-strand complementary DNA by reverse transcriptase Superscript III (Invitrogen). The amount of certain cDNA was measured in real-time PCR assays using a SYBR green Bio-Rad CFX96 system. Primers were: mouse α-SMA: sense 5′-CCCAGACATCAGGGAGTAATGG-3′, antisense 5′-TCTATCGGATACTTCAGCGTCA-3′; mouse CD90: sense 5′-TGCTCTCAGTCTTGCAGGTG-3′, antisense 5′-TGGATGGAGTTATCCTTGGTGTT-3′; mouse IGF-1: sense 5′-CACATCATGTCGTCTTCACACC-3′, antisense 5′-GGAAGCAACACTCATCCACAATG-3′; mouse IGF-2: sense 5′-GTGCTGCATCGCTGCTTAC-3′, antisense 5′-CGGTCCGAACAGACAAACT-3′; mouse PDGFA: sense 5′-TGGCTCGAAGTCAGATCCACA-3′, antisense 5′-TTCTCGGGCACATGGTTAATG-3′; mouse TGFβ-1: sense 5′-CCACCTGCAAGACCATGGAC-3′, antisense 5′-CTGGCGAGCCTTAGTTTGGAC-3′; mouse GM-CSF: sense 5′-GGCCTTGGAAGCATGTAGAGG-3′, antisense 5′-GGAGAACTCGTTAGAGACGACTT-3′; human IGF-1: sense 5′-ATGCTCTTCAGTTCGTGTGTG-3′, antisense 5′-GCACTCCCTCTACTTGCGTTC-3′; human IGF-2: sense 5′-GTGGCATCGTTGAGGAGTG-3′, antisense 5′-CACGTCCCTCTCGGACTTG-3′; human TGFβ-1: sense 5′-GGCCAGATCCTGTCCAAGC-3′, antisense 5′-GTGGGTTTCCACCATTAGCAC-3′; human GM-CSF: sense 5′-GGGAGCATGTGAATGCCATC-3′, antisense 5′-GCAGTGTCTCTACTCAGGTTCAG-3′. The expression level of the other genes was normalized to the expression of mouse GAPDH (sense 5′-AGGTCGGTGTGAACGGATTTG-3′, antisense 5′-TGTAGACCATGTAGTTGAGGTCA-3′) or human GAPDH (sense 5′-TGGCTTCATAGGTGACTTCCA-3′, antisense 5′-AAGGACCTGTCTAGGTTTGATGC-3′).

### Immunofluorescence (IF) staining

NFs with different treatments were seeded on 4-well chamber slides and fixed with 4% paraformaldehyde in PBS for 1 h at 15–25°C, washed with PBS. Then slides were incubated in permeabilisation solution (0.5% Triton X-100 in PBS) for 5 min. Following blockage with 5% BSA for 1 h samples were incubated with primary antibody (anti-mouse α-SMA antibody, Abcam #ab7817, 1:100) at 4°C overnight. After washing in 1× PBS, incubated with 1:200 diluted fluorescent secondary antibody for IF (Alexa 488 tagged). Signals were observed under fluorescence microscope.

### In vivo tumor studies

Male 6- to 8-week-old nude mice were used. Luciferase expressing CWR22Rv1 cells (1×10^6^), stably transfected by pCDNA3.0-luciferase plasmid, were mixed with Matrigel (1:1 in volume) and injected to the anterior lobes of 5 nude mice as control group. Eight mice were injected with CWR22Rv1 cells together with parental pshTERT cells (1:1). And another 8 mice were injected with CWR22Rv1 cells together with BM-MSCs pre-treated pshTERT cells. Six weeks after injection, mice were sacrificed and tumors were weighed. The research was approved and conducted following the rules and regulations of the University Committee of Animal Research (UCAR), No. 2002-296), which was fully credited by Association for Assessment and Accreditation of Laboratory Animal Care (AAALAC, No. A-3292-01), at the University of Rochester Medical Center.

### Statistical analysis

Values were expressed as mean ± standard deviation (SD). The Student’s t- and ANOVA tests were used to calculate p-values. p-values were two-sided, and considered statistically significant when <0.05.

## Results

### Identification of NFs and CAFs isolated from the PCa bearing mice

**Figure 1:**
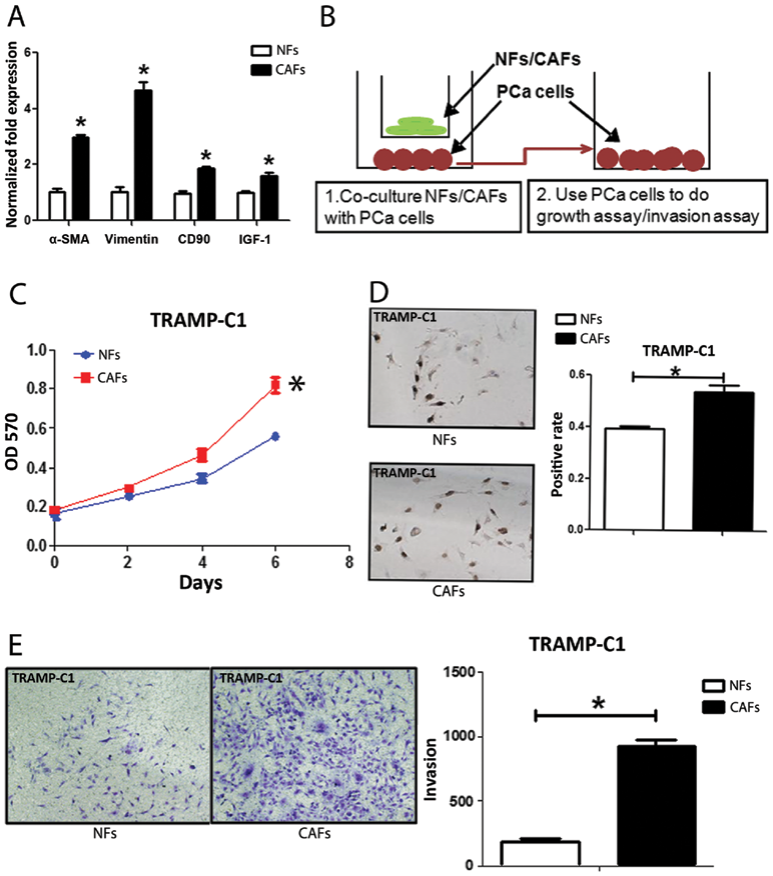
Identity of normal fibroblasts (NFs) and cancer associated fibroblasts (CAFs) isolated from the tumor bearing TRAMP mice. Characteristics of NFs and CAFs. NFs and CAFs were isolated from normal and cancer tissues and immortalized using SV40-T antigen incorporation. (A) Real-time PCR results show the obtained NFs and CAFs differentially express CAF specific markers, including CD90, IGF-1 and α-SMA. (B) Carton shows our protocols to do the growth and invasion assays. (C) The capabilities of NFs and CAFs to promote PCa cell growth are different in MTT assays. (D) BrdU staining shows the capability difference of NFs and CAFs to promote PCa cell growth. (E) The capabilities of NFs and CAFs to promote PCa cells invasion are different in transwell assays. ^*^p<0.05.

We first confirmed NFs and CAFs that were isolated from TRAMP mice with several markers for CAFs. The co-expression of α-SMA and vimentin is commonly used to define CAFs (12). CD90 (8) and IGF-1 (13) were also reported highly expressed in CAFs. We found the expression of α-SMA, vimentin, CD90 and IGF-1 in CAFs were higher in CAFs than NFs with qPCR (Fig. 1A), western blot analysis (Fig. 5D) and IF assays (Fig. 5E). We further compared their ability to enhance PCa cell growth and invasion as early reports documented that CAFs have much better capacity than NFs to enhance PCa cell growth and invasion (14). We co-cultured PCa epithelial cells with NFs vs CAFs in the co-culture systems (Fig. 1B), and results from MTT (Fig. 1C) and BrdU staining (Fig. 1D) assays revealed that the CAFs have better capacity than NFs to enhance PCa cells growth and proliferation. Furthermore, the results from the transwell invasion assay also showed that the CAFs had much better capacity than NFs to enhance PCa cell invasion (Fig. 1E).

Together, results from Fig. 1 confirmed the CAFs and NFs used in these studies are correct by showing CAFs have better capacity than NFs to enhance PCa cell growth and invasion.

### Molecular characteristics of NFs are changed upon co-culture with BM-MSCs

**Figure 2:**
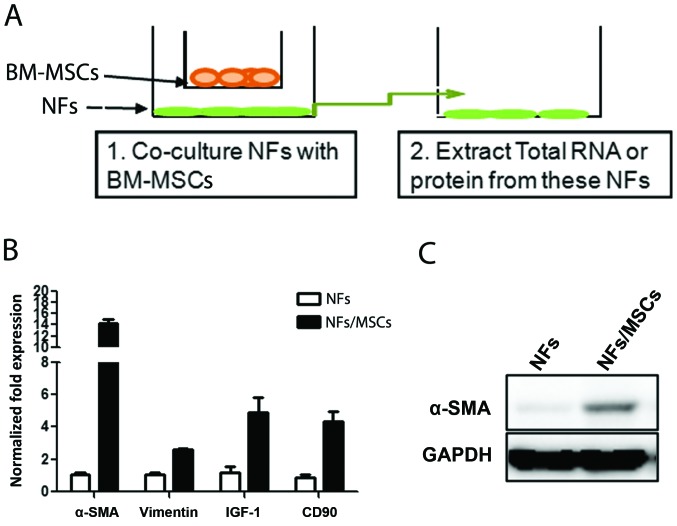
Co-culture of BM-MSCs and NFs changes the characteristics of NFs. The NF characteristics were altered when co-cultured with the BM-MSCs. NFs were co-cultured, with or without mouse primary BM-MSCs, which had been isolated from the normal B6 mice and cultured in transwell plates for 7 days. (A) The experimental protocol. (B) Total RNAs were then obtained from the NFs. The expression of CAF markers in NFs treated by BM-MSCs (MSCs) conditional media and control regular media were analyzed by real-time PCR. (C) Total proteins were extracted from NFs (BM-MSCs pretreated and control media-treated) for western blot analysis.

To investigate whether BM-MSCs have any influence on the conversion of prostate NFs to CAF cells, we co-cultured BM-MSCs with NFs (Fig. 2A) at a ratio of 1:10 for 7 days, and results from qPCR and western blot analyses revealed increased CAF markers, including α-SMA, CD90, and IGF-1 in NFs upon co-culture with BM-MSCs (Fig. 2B and C). Similar results were obtained when we replaced the ratio of BM-MSCs with NFs from 1:1 to 1:2 or 1:4 (data not shown). These results suggest that BM-MSCs can influence the conversion of NFs to cells with characteristics more close to CAFs.

### NFs promote PCa cell invasion when pre-treated with BM-MSCs

**Figure 3:**
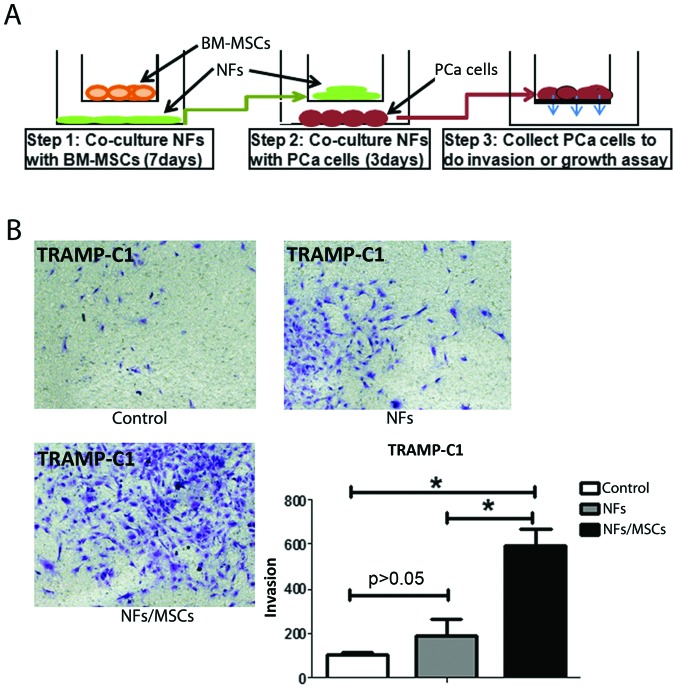
NFs promote PCa cell invasion when pre-treated with BM-MSCs. (A) The strategy of performing PCa cell invasion test. NFs (mouse NF or human pshTERT cells) were co-cultured with mouse or human BM-MSCs for 7 days, respectively. The NFs were subsequently incubated with PCa cells for 3 days, and then the PCa cells were harvested, and their invasion ability was tested in transwell plates. (B) The invasion ability of TRAMP-C1 cells co-cultured with NFs or BM-MSCs (MSCs)-treated NFs were tested. Quantitation shown on the right. ^*^p<0.05.

One of the key phenotypes of CAFs is their capability to promote tumor metastasis (Fig. 1E) (2). Our next experiment was to test whether BM-MSCs-treated NFs also would have a similar capability. After co-culturing mouse BM-MSCs and mouse NFs for 7 days, we removed the mouse BM-MSCs (to eliminate the BM-MSCs effect on PCa cells) and collected mouse NFs. These pre-treated NFs were subsequently incubated with the mouse PCa epithelial TRAMP-C1 cells (at the ratio of 1:1) for 3 days (see detailed procedure in Fig. 3A). The results showed that NFs pre-treated/incubated with BM-MSCs have the capacity to enhance the PCa cell invasion (Fig. 3B).

Similar results were obtained when we replaced mouse BM-MSCs and mouse NFs with human BM-MSCs and human stromal pshTERT cells showing pshTERT cells (after co-cultured with human BM-MSCs) were able to promote PCa C4-2 (Fig. 1) and LNCaP cells invasion (Fig. 6B).

The above, together with the results in Fig. 3 suggest that the prostate NFs may acquire CAFs characteristics via co-culture with BM-MSCs to enhance PCa cell invasion.

### NFs promote PCa cell growth when pre-treated with BM-MSCs

**Figure 4:**
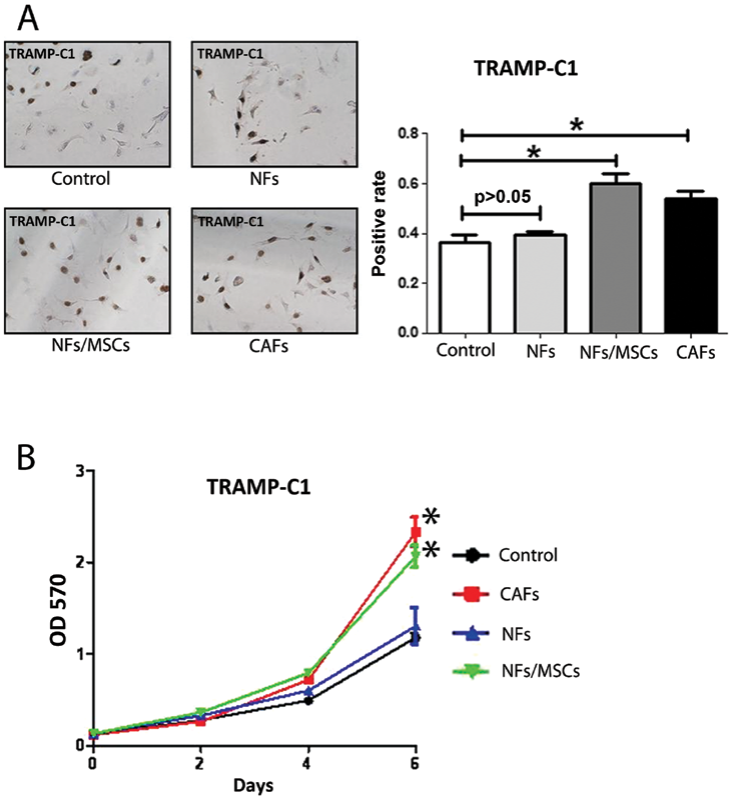
The BM-MSC pre-treated NFs promote PCa cells growth. Growth assay follows the protocol demonstrated in Fig. 3A, similar to invasion assay. NFs were co-cultured with the primary mouse BM-MSCs for 7 days, subsequently incubated with PCa cells for 3 days, which were harvested for the growth assay. (A) The growth of TRAMP-C1 cells co-cultured with NFs, BM-MSCs pre-treated NFs, CAFs and control media was demonstrated by BrdU staining, the quantitation of growth rates are shown on the right. (B) The viability of TRAMP-C1 cells in different treatments were tested in MTT assay. Quantitation is shown on the right. ^*^p<0.05.

Another key phenotype of CAFs is their capability to promote tumor growth (2). Using BrdU staining assay and MTT assay to detect PCa cells proliferation (Fig. 4A) and growth (Fig. 4B), we found that the NF pre-treated BM-MSCs increased the PCa cells growth (Fig. 4A) and proliferation (Fig. 4B). *In vivo* mouse studies using mice with orthotopically xenografted CWR22Rv1 cells co-implanted with NF cells pre-treated with either BM-MSCs or control media for 7 days also showed that NFs pre-treated with BM-MSCs have larger tumor size as compared to the media control (Fig. 6A).

The *in vitro* and *in vivo* results from Figs. 4 and 6A suggest that the prostate NFs may acquire the CAF characteristics via co-culture with BM-MSCs to enhance PCa cell growth and proliferation.

### BM-MSCs secrete TFGβ-1 to promote the conversion from NFs to CAFs

**Figure 5:**
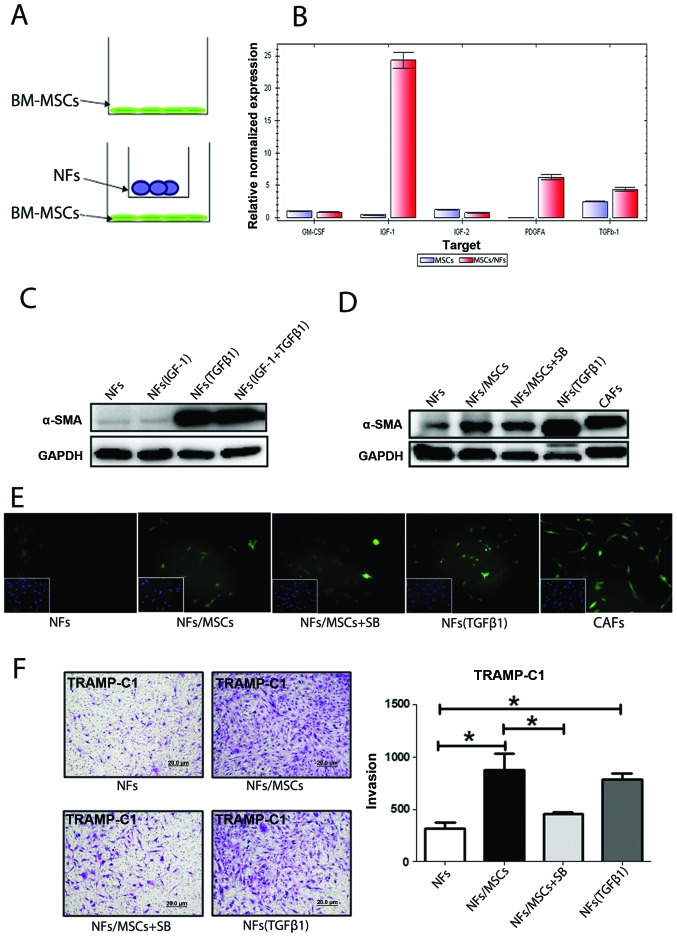
Mechanism by which BM-MSCs alter the NF characteristics. (A) The strategy of extracting total RNA from BM-MSCs after co-culture with NFs. or BM-MSCs alone, which were used as a control. (B) Real-time PCR assay shows TGF-β1, IGF-1 and PDGFA mRNA expression levels are increased in mouse BM-MSCs upon co-cultured with mouse NFs. (C) For western blot assay, NF cells were treated with TGF-β1 or (and) IGF-1 for 3 days, then total protein from NFs was extracted. The protein expression of α-SMA in NFs, the major marker of CAF, was increased after treated with TGF-β1 but not IGF-1. (D) When inhibitor of TGF-β1 was added into the co-culture system, α-SMA was lightly decreased, showing the NFs to CAFs conversion was partially blocked. (E) Immunofluorescence results showing the expression of α-SMA after different treatments. (F) Co-cultured TRAMP-C1 cells with differently pre-treated NFs. The TRAMP-C1 cells were collected for the invasion assay. Quantitation is shown on the right. ^*^p<0.05.

**Figure 6:**
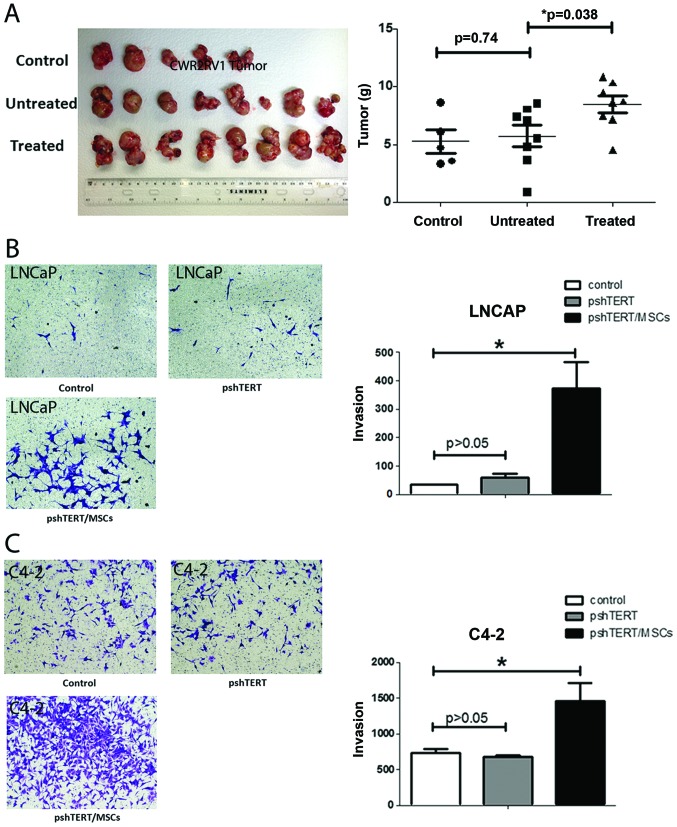
The BM-MSCs pre-treated NFs promote human PCa tumor growth and PCa cell invasion. (A) CWR22Rv1 cells, either alone or together with untreated or BM-MSCs 7-day pre-treated pshTERT cells were orthotopically injected into nude mice. Mouse group 1, CWR22Rv1 alone; group 2, CWR22Rv1 cells + pshTERT cells (media treated, control); group 3, CWR22R1 + pshTERT cells (pre-treated with hBM-MSCs for 7 days). For groups 2 and 3, the mixtures of CWR22Rv1 and pshTERT cells in a ratio of 5:1 were used in injection. After 6 weeks, mice were sacrificed and tumor weight was analyzed. Quantitation is shown on the right. ^*^p<0.05. (B) Images of the invasion ability of LNCaP cells co-cultured with pshTERT or human BM-MSCs treated pshTERT cells. (C) Images of the invasion ability of C4-2 cells co-cultured with pshTERT or human BM-MSCs treated pshTERT cells.

Recent studies demonstrated that some cytokines or growth factors including GM-CSF, IGF-1, IGF-2, PDGFA and TFGβ-1 induced the conversion of NFs to CAFs (15–17). To investigate which growth factors or cytokines secreted by BM-MSCs might induce this transformation, we extracted RNAs from BM-MSCs, with or without co-culture with NFs (Fig. 5A), to assay their differential expression of these growth factors/cytokines. The qPCR analyses revealed that expression of PDGFa, IGF-1 and TGFβ-1 in BM-MSCs was increased after co-culture with NFs (Fig. 5B). Similar results with increased IGF-1 and TGFβ-1 also occurred when we replaced mouse BM-MSCs/NFs cells with human BM-MSCs/NFs cells PCa (data not shown).

We then applied two different approaches to further confirm these results: first we added IGF-1 and TGFβ-1 recombinant proteins to NFs to mimic the BM-MSCs effect and found only TGFβ-1 induced expression of α-SMA (Fig. 5C). We then used an interruption approach via addition of TGFβ-1 inhibitor SB431542 to the co-cultured NFs and BM-MSCs and found slightly suppressed α-SMA expression (Fig. 5D and E). Importantly, addition of the TGFβ-1 inhibitor SB431542 suppressed the BM-MSCs/NFs induced PCa cells invasion (Fig. 5F).

Results in Fig. 5 suggest that BM-MSCs may be able to trigger the conversion of NFs to CAFs via altering the secretion of TGFβ-1.

## Discussion

PCa is the most common malignant tumor of men in the USA with the second highest death rate (1). Even though the current standard therapy of androgen deprivation therapy (ADT) for the later stage PCa is effective in the beginning, eventually the PCa still progresses into the castration resistant stage with metastasis (18). Several hypotheses were used to explain the mechanisms by which PCa could escape from ADT (19), yet none of them was applied successfully to cure PCa. It is now widely recognized that PCa, like other tumors, is regulated by multiple signaling from multiple cells existing in the TME, including luminal epithelial cells (19), epithelial basal cells, stromal cells, stem/progenital cells (20), BM-MSCs (9), endothelial cells (21), and macrophages (22).

The stromal CAFs in TME, were also reported to be able to influence the PCa progression (2). However, the origin of CAFs remained unclear. Early studies suggested that the most important source of CAFs could come from the resident NFs (17), especially the spindle-like fibroblasts surrounding tumors (so-called peritumor fibroblasts), and not the fibroblasts far away from tumor sites, with distinct characteristics of highly expressed α-SMA, CD90, IGF-1 (8,23,24) that can promote tumor growth and invasion (7,14). The second source of CAFs could come from the endothelial-mesenchymal transition (EndMT) (25) with increased mesenchymal markers of fibroblast-specific protein-1 (FSP1) and decreased CD31/PECAM (25). Importantly, BM-MSCs were reported to be the potential third source of CAFs (26) that might contribute ~25% of the CAF population (27).

The bone marrow is a complex tissue containing hematopoietic stem/progenitor cells and their connective tissue mesenchymal cells constituting the bone marrow microenvironment. BM-MSCs comprise multifunctional non-hematopoietic stem cells that have differentiating capabilities (28). In mouse models, BM-MSCs were demonstrated to be able to migrate and incorporate into other tissues where BM-MSCs were able to elicit tissue-specific differentiation. For example, BM-MSCs contributed to tissue repair by differentiation into tissue-specific cell types or by production of trophic factors at the site of injury to stimulate tissue repair and/or to reduce self-inflicted damage mediated by the immune system (29). In PCa, BM-MSCs were also proven to be able to migrate to PCa and influence PCa cell growth (30), and invasion with increased PCa stem cell population (9).

We found, for the first time, that BM-MSCs, in addition to differentiating to CAFs or their direct function on PCa epithelial cells, they might also be able to promote the conversion of NFs to CAFs via secretion of TGFβ-1. These results suggest that the recruited BM-MSCs may be able to transform into CAFs and further promote the conversion of normal fibroblasts to CAFs. The consequences of such increased CAFs may then enhance the PCa cell growth and invasion.

The significance of the above findings is not only adding new evidence of cross-talk between different cell types within the TME, it also provides a new potential target to suppress PCa progression. Targeting the infiltrating BM-MSCs via either interruption of interaction between PCa and BM-MSCs or preventing the conversion of NFs to CAFs via inhibition of TGFβ-1 signal may result in suppression of the PCa progression.
